# Supplementary figures and images for: Artifactual pyrosequencing reads in multiple-displacement-amplified sediment metagenomes from the Red Sea
Source: PeerJ. 2013 Apr 30;1:e69. doi: 10.7717/peerj.69 (PMC3642703; doi:10.7717/peerj.69)

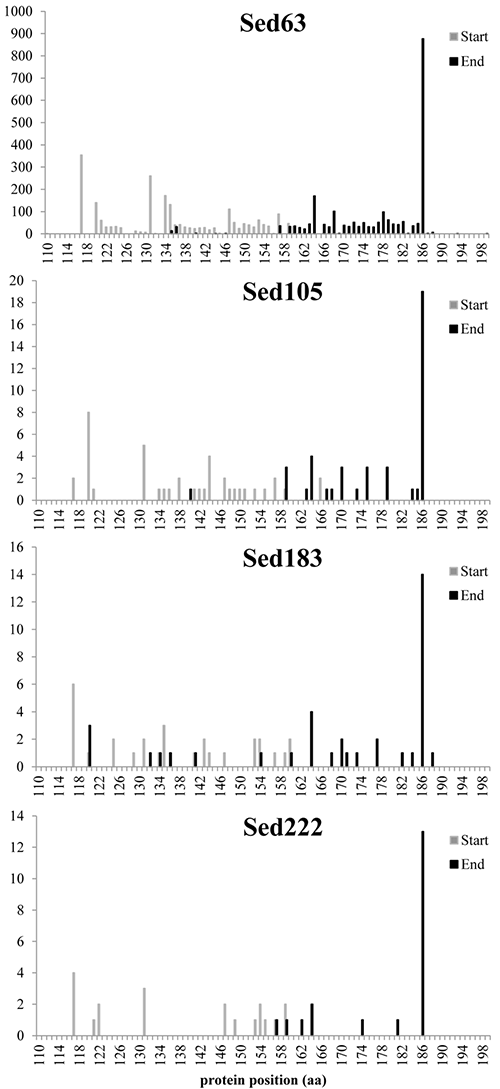

Supplement: Figure S1 — Start and end positions of the BLASTX result for K06988 protein were extracted. The figure shows the distribution of start and end positions of reads. [file peerj-01-69-s001.png]

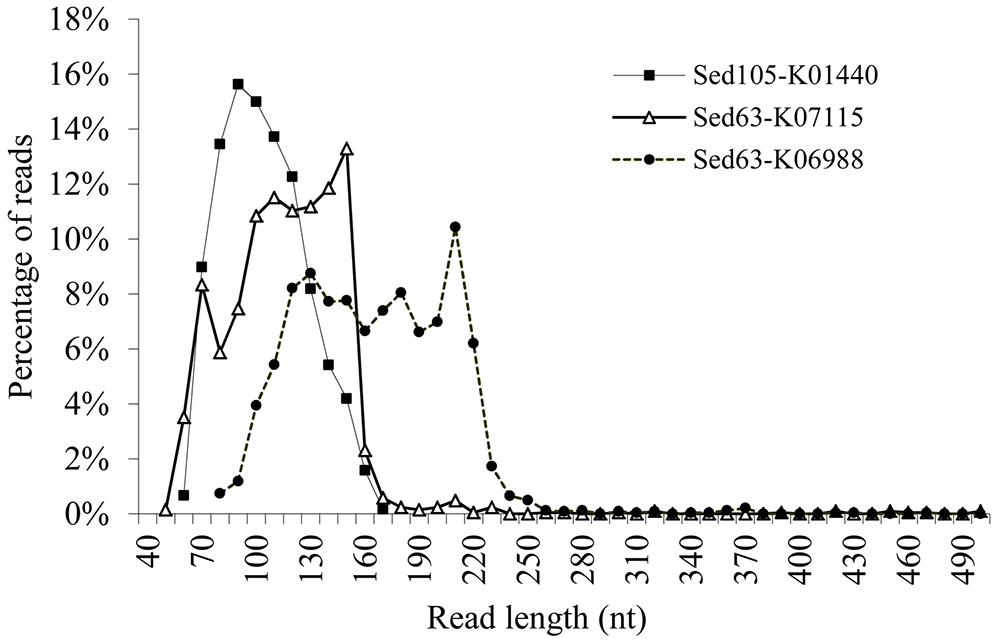

Supplement: Figure S2 — (A) K06988 and (B) K00984 [file peerj-01-69-s002.png]

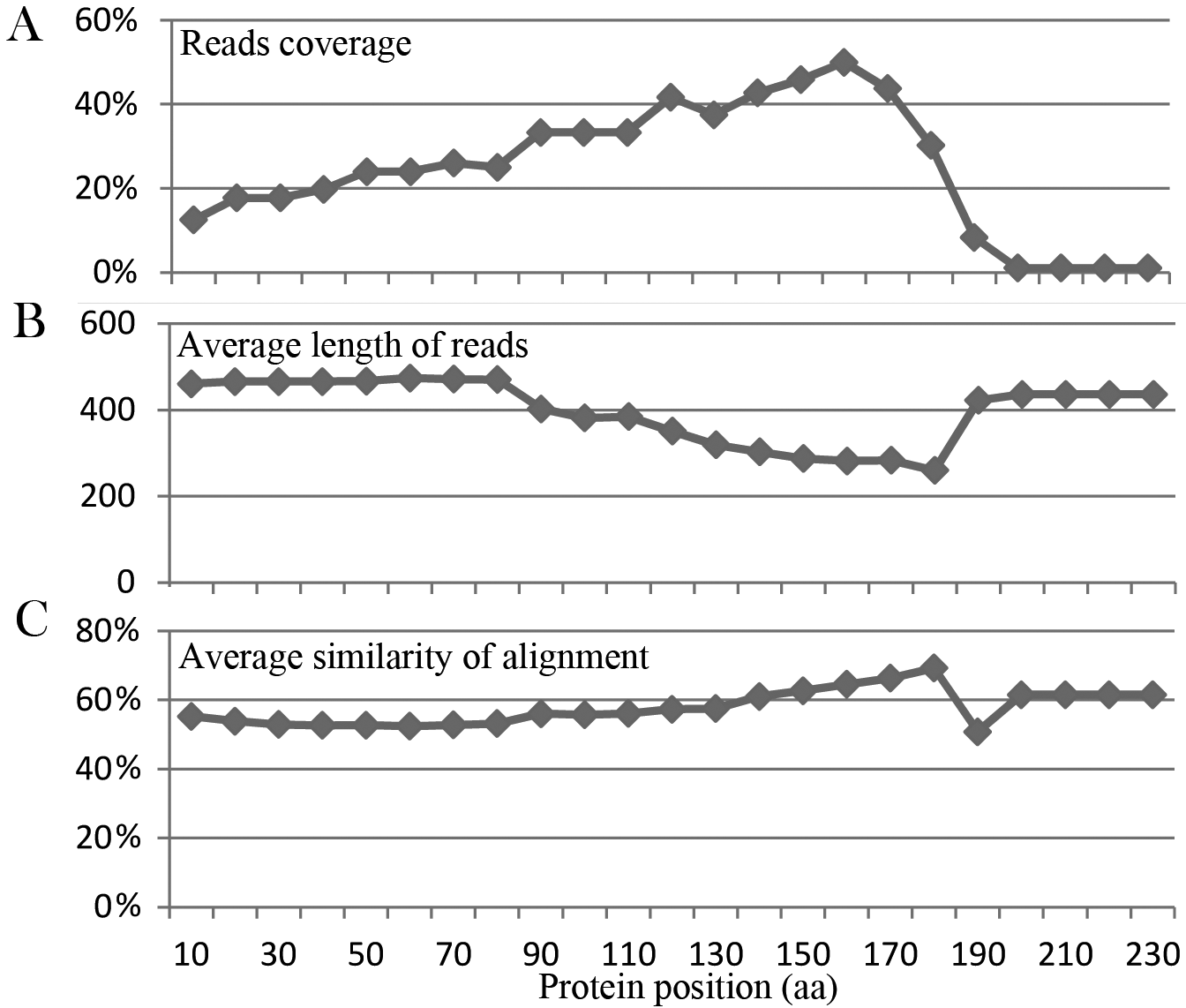

Supplement: Figure S3 [file peerj-01-69-s003.png]

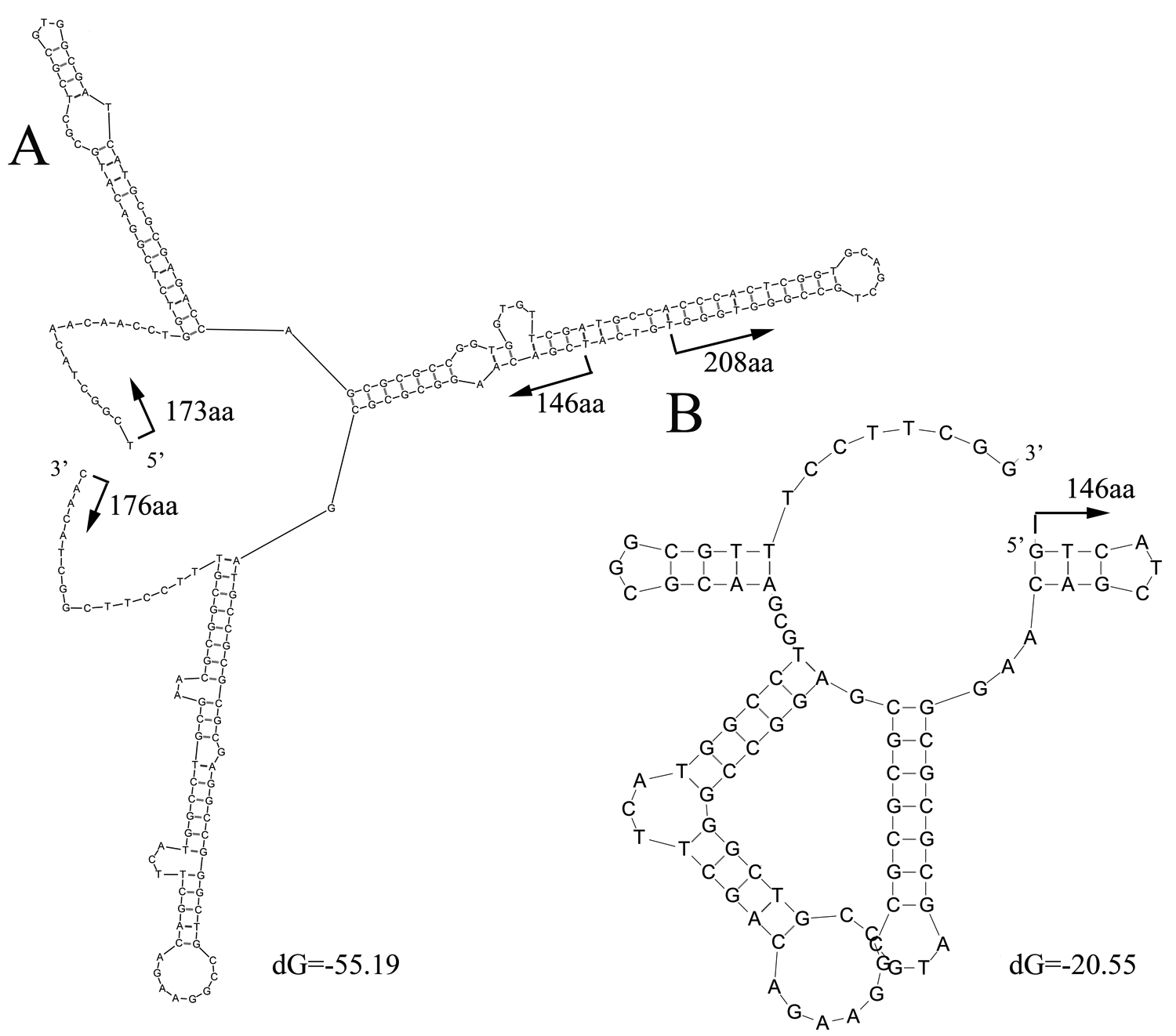

Supplement: Figure S4 — The two selected internal regions were where most of the reads were matched. Protein positions of KDO 8-P synthase (K01627) and 6-phosphogluconate dehydrogenase (K06988) were labeled to show hotspots of the alignments between the reads and proteins. [file peerj-01-69-s004.png]

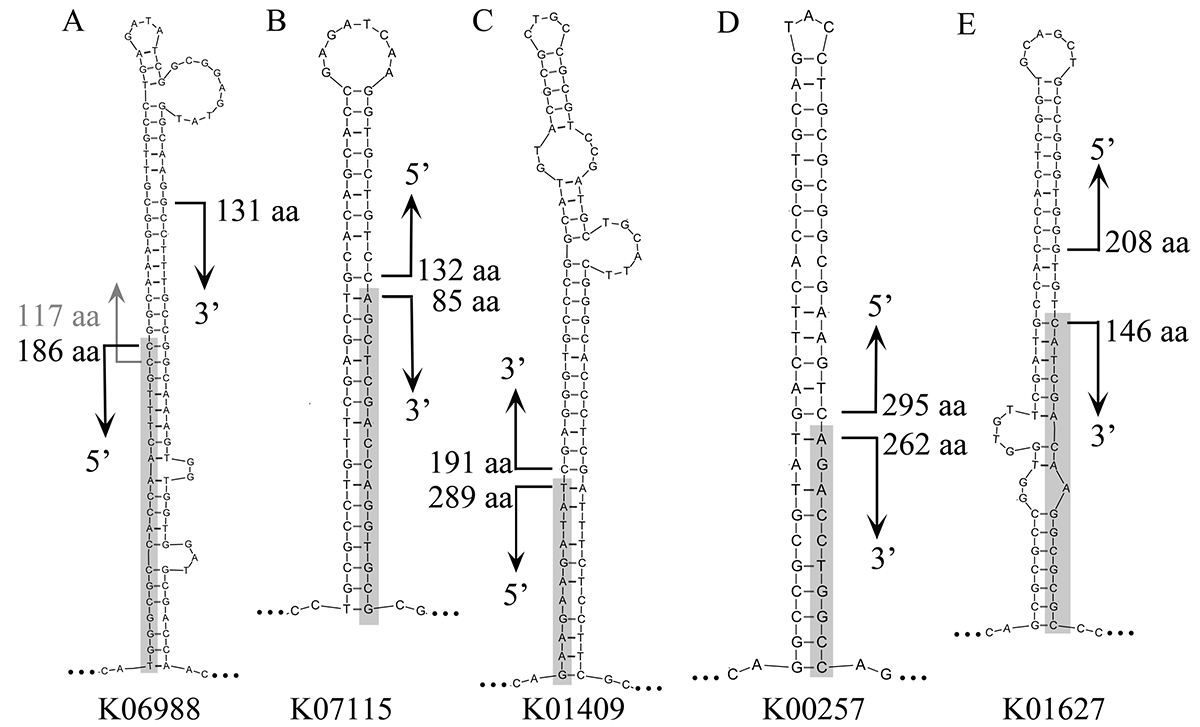

Supplement: Figure S5 — Alignment start and end hotspots in the BLASTX results of five selected genes were labeled on the stems. The translocated fragments supposed to attach to the stems were shaded. [file peerj-01-69-s005.png]
