# Supplementary material for: Artifactual pyrosequencing reads in multiple-displacement-amplified sediment metagenomes from the Red Sea
Source: PeerJ. 2013 Apr 30;1:e69. doi: 10.7717/peerj.69 (PMC3642703; doi:10.7717/peerj.69)
Supplement: Table S1 — Abbreviations for species names are as following. Reu: Ralstonia eutropha JMP134; rpi: Ralstonia pickettii; rme: Cupriavidus metallidurans; reh: Ralstonia eutropha H16; cti: Cupriavidus taiwanensis; vap: Variovorax paradoxus; bac: Burkholderia ambifaria. [file peerj-01-69-s006.doc]

Table S1 Most frequent alignment start and stop positions between the reads and proteins from the species.

Abbreviations for species names are as following. Reu: *Ralstonia eutropha* JMP134; rpi: *Ralstonia pickettii*; rme: *Cupriavidus metallidurans*; reh: *Ralstonia eutropha* H16; cti: *Cupriavidus taiwanensis*; vap: *Variovorax paradoxus*; bac: *Burkholderia ambifaria*.

|  |  |  | Hotspots (aa) | |
| --- | --- | --- | --- | --- |
| Gene | Layer | Species | Start | Stop |
| K06988 | Sed63 | reu | 131 | 186 |
| K07115 | Sed63 | rpi,rme | 86 | 138 |
| K01440 | Sed105 | rme,reh | 121,134 | 165,175 |
| K01409 | Sed105 | reu, reh | 191 | 289 |
| K00257 | Sed183 | cti | 255,260 | 299 |
| K01627 | Sed222 | cti,vap,bac | 146 | 206,208 |
