# Supplementary material for: Artifactual pyrosequencing reads in multiple-displacement-amplified sediment metagenomes from the Red Sea
Source: PeerJ. 2013 Apr 30;1:e69. doi: 10.7717/peerj.69 (PMC3642703; doi:10.7717/peerj.69)
Supplement: Table S2 [file peerj-01-69-s007.doc]

Table S2 Free energy of short reads for K06988 and random short reads.

Average length of the random sequences was about 100 bp, 120 bp, 140 bp, and 160 bp. That of the random sequences for K00984 was 160 bp and that of K06988 for Sed63 was 162.

|  | Sed12 | Sed63 | Sed105 | Sed183 | Sed222 |
| --- | --- | --- | --- | --- | --- |
| K06988 | -- | -42.7(14.3) | -37.8 (11.3) | -41.2(15.3) | -45.7(16.3) |
| Random_100 | -11.0(6.7) | -13.8(7.0) | -14.6(7.0) | -15.8(7.0) | -13.3(6.6) |
| Random_120 | -13.7(8.5) | -16.8(9.2) | -18.2(9.2) | -19.9(8.6) | -16.4(9.1) |
| Random_140 | -16.3(9.2) | -19.5(9.7) | -21.6(10.0) | -23.3(9.2) | -19.4(10.4) |
| Random_160 | -19.0 (10.4) | -22.9(10.6) | -24.5(10.7) | -26.6(10) | -21.1(10.9) |
| K00984_random | -15(4.9) | -15(5.3) | -15.7(5.3) | -15.2(5.3) | -15.3(5.4) |
